# Comparison of routine del Nido cardioplegia vs two types of modified del Nido cardioplegias for myocardial protection among patients undergoing coronary artery bypass grafting (CABG) surgeries: A randomized double-blind clinical trial

**DOI:** 10.1051/ject/2024011

**Published:** 2024-09-20

**Authors:** Babar Ali, Salman Pervaiz Butt, Mohammad Ghazi Nour, Mohammad Bagher Khosravi, Naeimehossadat Asmarian, Ali Raza Shoul, Arun Kumar, Umer Darr, Gopal Bhatnagar

**Affiliations:** 1 Student Research Committee of Shiraz University of Medical Sciences PO BOX 71348-14336 Shiraz Iran; 2 Heart Vascular and Thoracic Institute, Cleveland Clinic Abu Dhabi PO BOX 112412 United Arab Emirates; 3 Department of Surgery, Section of Cardiac Surgery, Shiraz University of Medical Sciences PO BOX 71348-14336 Shiraz Iran; 4 Anesthesiology and Critical Care Research Center, Shiraz University of Medical Sciences PO BOX 71348-14336 Shiraz Iran; 5 Department of Perfusion, Division of Anesthesiology and Critical Care Research Center, Shiraz University of Medical Sciences PO BOX 71348-14336 Shiraz Iran

**Keywords:** Myocardial protection, Modified del Nido cardioplegia solution, Coronary artery bypass grafting (CABG) surgery

## Abstract

*Background*: The del Nido cardioplegia solution is a widely used method for myocardial protection in various settings. However, there is limited evidence of its effectiveness in adult cardiac surgery, and the baseline solution, Plasma Lyte A, is not readily available, leading to the use of alternative baseline solutions. This study aims to investigate the effectiveness of routine del Nido cardioplegia in adult cardiac surgery and the impact of different baseline solutions on myocardial protection and other perioperative outcomes. *Methods*: This study was a prospective, double-blind randomized parallel group clinical trial conducted at a single tertiary care hospital in Iran. A total of 187 adult patients were evaluated for eligibility, of which 120 met the inclusion criteria for elective isolated CABG surgery. The patients were randomly assigned to three groups, with each group consisting of 40 patients. The control group received a normal saline-based routine del Nido cardioplegia, Intervention Group A received Ringer lactate-based del Nido cardioplegia, and Intervention Group B received plain Ringer-based del Nido cardioplegia. The levels of Creatine Kinase-MB (CK-MB), Troponin T, Troponin I, and lactate were primarily assessed at four different times: after anesthesia induction (Baseline), 2 h, 12 h, and 24 h. *Results*: Preoperative demographic and clinical characteristics were the same among groups with insignificant differences (*p* > 0.05). There was no significant difference among groups based on CK-MB, Troponin T, Troponin I, and lactate levels (*p* = 0.078, 0.143, 0.311, and 0.129 respectively). However, there was a significant difference in the time effect of Troponin T and Lactate (*p* = 0.034, *p* = <0.001). *Conclusion*: Normal saline, Ringer lactate, and plain Ringer provide comparable myocardial protection in adult-isolated CABG surgery with modified del Nido cardioplegia. Larger studies are needed to identify the best alternative to Plasma Lyte A while maintaining del Nido cardioplegia as the control.

## Introduction

In most cardiovascular surgical procedures, surgeons require a still and without blood flow heart to perform the procedure accurately and safely. To achieve this, the heart is stopped and bypassed for a specific period, depending on the type of surgery [1]. One such surgery, coronary artery bypass grafting (CABG), is performed frequently worldwide due to the high prevalence of coronary artery disease (CAD) in both developed and underdeveloped countries and also requires a bloodless and static field in most cases [2–4]. During cardiac surgery, protecting the myocardium is crucial to prevent complications such as ischemic reperfusion injury, myocardial infarction, and low output syndrome [5]. Since the 1950s researchers worldwide have been working to develop the best approach for myocardial protection during surgery. Intracellular or extracellular cardioplegia in either blood or crystalloid solution has been used in many setups to protect the myocardium. However, each formulation has some undesirable effects, especially in pediatric cases [6]. In the early 1990s, researchers from the University of Pittsburgh developed a cardioplegia solution specifically tailored to meet the unique needs of immature hearts. Their focus was on addressing intracellular calcium, myocardial high-energy phosphates, lactate production, and intracellular buffering. Subsequently, Dr. Pedro Del Nido further refined and modified the solution for myocardial protection, with a particular emphasis on immature pediatric myocardium. Boston Children’s Hospital has been successfully employing this solution for more than two decades now, with positive outcomes in congenital cases. Since 2003, this solution has also been used effectively in adult cardiac surgery. The solution’s efficacy and safety make it a promising option for cardiac surgery in both adult and pediatric populations [7, 8]. Del Nido cardioplegia has also been proven effective in adult minimal invasive cardiac surgeries and acquired cases (coronary artery bypass, valvular, and combined surgeries) [9–11].

Mick and Sevuk et al. have found that del Nido solution, when compared to other cardioplegias or when modified with different additives, can have beneficial effects over traditional cardioplegia [12, 22]. Another study by Karaarslan et al. showed that del Nido cardioplegia is an effective and cost-efficient option for coronary surgery with similar clinical outcomes to traditional blood cardioplegia [13]. Lactated Ringer-based del Nido solution can also provide myocardial protection comparable to the blood cardioplegia strategy [14]. Another study has explored modifications to del Nido cardioplegia, with promising results in preserving LV (Left Ventricle) function and inducing spontaneous sinus rhythm return [15, 16]. A meta-analysis suggests that del Nido cardioplegia may be associated with lower perioperative mortality than Custodial or Blood Cardioplegia in adult patients, but the risk of atrial fibrillation may be lower with Custodial Histidine-tryptophan-ketoglutarate (HTK) [17]. However, there has been no study comparing the modified version of the del Nido cardioplegia solution to other modified versions of the del Nido cardioplegia solution, making this study the first of its kind to our knowledge. At our center (Shahid Faghihi Hospital), we use a modified version of del Nido cardioplegia called routine del Nido cardioplegia solution (rdNCS) for both adult and pediatric cardiac surgeries. For the past 8 years, we have been using 0.9% normal saline as the baseline solution instead of Plasma Lyte A, which is limitedly available in certain Asian countries, including Iran.

Therefore, this study aims to provide more definitive evidence on the superiority of one solution over others based on various outcomes, including Creatine Kinase-MB (CK-MB) level, Troponin level, bypass time, clamp time, incidence of ventricular fibrillation, postoperative atrial flutter, atrial fibrillation, ventilation support time, Intensive Care Unit (ICU) stay, and perioperative mortality. The study is particularly focused on the use of del Nido cardioplegia as a myocardial protection solution for adult surgeries and the composition of the solution. In addition, this study also hypothesized that the Ringer lactate-based del Nido cardioplegia solution performs better than normal saline and plain Ringer-based del Nido cardioplegia solutions. The findings of this study could contribute to clinical practice and improve patient outcomes in CABG surgeries.

## Methods and materials

### Trial design and setting

This study was conducted at a single tertiary care hospital in Iran from February 1 to June 20, 2023. This was a prospective, double-blinded, randomized, parallel-group clinical trial.

### Participants

A total of 187 adult patients were evaluated for eligibility, of which 120 met the inclusion criteria. Patients more than 18 years old with a minimum ejection fraction of 30%, diagnosed with triple vessel coronary artery disease, and scheduled for on-pump elective isolated CABG surgery were included and randomly assigned to three groups. The Control Group received rdNCS made with normal saline (*n* = 40). Intervention Group A received a modified del Nido Cardioplegia Solution (rldNCS) made with Ringer lactate (*n* = 40). Intervention Group B received a modified del Nido Cardioplegia Solution (prdNCS) made with plain Ringer (*n* = 40). The effectiveness of these interventions was evaluated through follow-up assessments of the participants.

### Procedure

All patients underwent the standard protocol for general anesthesia, with routine drugs used for induction and maintenance. The surgical approach involved a median sternotomy along with graft preparation and a cardiopulmonary bypass was established by connecting an arterial cannula to the ascending aorta for arterial limb access, and a two-stage cannula was connected to the right atrium for venous drainage via the venous limb. The cardioplegic arrest was induced by cardioplegia after aortic cross-clamping at the core body temperature of 32–34 °C.

### Intervention

The cardioplegic solutions were given at the aortic root after cross-clamping for myocardial protection via the antegrade route using standardized aseptic techniques routinely used as per hospital policy. The solutions were prepared by the perfusionist according to the instructions present in a sealed envelope. The rdNCS made with normal saline was modified at the service moments before their administration to the myocardium via aortic root, and either the Ringer lactate (modified del Nido I or Intervention A) or plain Ringer’s (modified del Nido II or Intervention B) used as the crystalloid baseline solution per randomization received in an opaque envelope. The autologous blood and crystalloid ratio were 1:4. The rdNCS composition comprises normal saline 0.9% 800 mL (Baseline Solution), containing Sodium (Na+) 154 mEq/L, Chloride (Cl−) 154 mEq/L, and Tonicity 308 mOsm/L, with a pH of approximately 5.5. This solution also includes additives such as Mannitol 20% 16.3 mL, Magnesium sulfate 50% 4 mL, Sodium bicarbonate 8.4% (1 mEq/mL) 20 mL, Potassium chloride (2 mEq/mL) 13 mL, and Lidocaine 1% 13 mL. The bicarbonate level of rdNCS is 20 mL, which is slightly higher than the typical level of 13 mL in original del Nido cardioplegia. This increase is necessary to maintain the solution’s acidic pH at a somewhat basic level. Elevated bicarbonate levels can elevate the risk of post-clamp-off arrhythmia and the need for defibrillation. However, the Lidocaine component of del Nido cardioplegia mitigates these risks by helping to control such arrhythmias.

On the other hand, the rldNCS composition comprises lactated Ringers 800 mL, containing Sodium (Na+) 130 mEq/L, Chloride (Cl−) 109 mEq/L, Potassium (K+) 4 mEq/L, Calcium (Ca++) 1.5 mEq/L, Magnesium (Mg++) Lactate 28 mEq/L, and Tonicity 276 mOsm/L (Hypotonic), with a pH of approximately 6.5. This solution also includes additives such as Mannitol 20% 16.3 mL, Magnesium sulfate 50% 4 mL, Sodium bicarbonate 8.4% (1 mEq/mL) 13 mL, Potassium chloride (2 mEq/mL) 13 mL, and Lidocaine 1% 13 mL.

Finally, the prdNCS composition comprises plain Ringers 800 mL, containing Sodium (Na+) with 147 mEq/L Chloride (Cl−) 155 mEq/L, Potassium (K+) 4 mEq/L, Calcium (Ca++) 4 mEq/L, and Tonicity 311.3 mOsmol/L, with a pH of approximately 7.4. This solution also includes additives such as Mannitol 20% 16.3 mL, Magnesium sulfate 50% 4 mL, Sodium bicarbonate 8.4% (1 mEq/mL) 13 mL, Potassium chloride (2 mEq/mL) 13 mL, and Lidocaine 1% 13 mL.

The composition of each solution can also be observed in Table 1. The cardioplegia solutions were administered through a single dose of 20 mL/kg maximum of 1000 mL for patients weighing more than 50 kg. A single dose of del Nido cardioplegia provides adequate myocardial protection for 90 min and reduces the myocardial temperature to less than 15 °C, minimizing oxygen consumption. However, 3/4 (75%) of the dose is necessary for effective myocardial protection [18]. The usual delivery temperature of cardioplegia solution was 4 °C, system pressure of 100–200 mm Hg, and pump flow was 200–300 mL/min [19]. No additional doses were infused among participants.

**Table 1:** Composition of routine and modified del Nido cardioplegia.

del Nido Cardioplegia 1:4 (Blood: Crystalloid)					
Routine dNCS (rdNCS)		Modified I dNCS (rldNCS)		Modified II dNCS (prdNCS)	
Normal saline 0.9%	800 mL	Ringer lactate	800 mL	Plain Ringer	800 mL
Sodium-bicarbonate 1 mEq/mL	20 mL	Sodium-bicarbonate 1 mEq/mL	13 mL	Sodium-bicarbonate 1 mEq/mL	13 mL
Mannitol (20%)	16.3 mL	Mannitol (20%)	16.3 mL	Mannitol (20%)	16.3 mL
Magnesium sulfate (50%)	4 mL	Magnesium sulfate (50%)	4 mL	Magnesium sulfate (50%)	4 mL
Lidocaine (1%)	13 mL	Lidocaine (1%)	13 mL	Lidocaine (1%)	13 mL
Potassium-chloride 2 mEq/mL	13 mL	Potassium-chloride 2 mEq/mL	13 mL	Potassium-chloride 2 mEq/mL	13 mL
Blood	200 mL	Blood	200 mL	Blood	200 mL

### Laboratory assessment

During the study, arterial blood was collected from patients at four different times – after anesthesia induction (baseline), 2 h, 12 h, and 24 h post-surgery. The collected blood samples were then used to conduct serum CK-MB, Troponin T, Troponin I, and lactate tests. The CK-MB enzyme is predominantly present in cardiac muscle cells and is vital for their energy production process. In the event of heart muscle damage, such as during a heart attack, CK-MB is released into the bloodstream. The typical CK-MB range is between 5 and 25 U/L. Troponin T is an essential protein present in heart muscle cells that is responsible for regulating the contraction mechanism of the cardiac muscle. It is a crucial component of the troponin complex that oversees the heart’s muscle contractions. In the event of heart muscle damage, such as during a myocardial infarction (heart attack), Troponin T is released into the bloodstream. The typical range for Troponin T is below 0.01 ng/mL. Troponin I is a vital protein present in the heart muscle that plays a crucial role in regulating cardiac muscle contraction. It is exclusively found in the heart and is released into the bloodstream when heart muscle cells sustain damage. The normal value of Troponin I stands at <0.04 ng/mL. Lactate, which is also referred to as lactic acid, is a natural byproduct of the body’s anaerobic metabolism. This process occurs when glucose is broken down in the absence of sufficient oxygen levels. Lactate is an alternative pathway for energy production that the cells must resort to under such conditions. The normal range for lactate is 4.5–19.8 mg/dL. The hospital’s clinical diagnostic laboratory performed the measurements, ensuring that their devices were regularly checked and calibrated as part of their daily practice. The laboratory staff was kept unaware of the patients’ assigned groups. The study assessed myocardial protection by examining the increase in cardiac biomarkers in the presence of Electrocardiogram (ECG) abnormalities or symptoms. Along with the laboratory assessment team of researchers closely monitored the surgical and clinical outcomes during the perioperative period and hospital stay.

### Clinical outcomes

The primary outcomes were assessed for myocardial protection between routine and modified del Nido cardioplegia solution using the serum levels of cardiac enzymes including CK isoenzyme MB (CK-MB), Troponin T, Troponin I, and lactate in the immediate postoperative period 2 h as well as 12 h and 24 h, postoperatively. It’s important to note that cardiac markers, including CK-MB, Troponin T, Troponin I, and lactate, were not monitored during the surgery because the heart is typically bypassed during this period. Consequently, the patient’s blood is diverted to a reservoir, leading to dilution and potentially altering the true values of these cardiac markers. The secondary outcomes include intraoperative assessments of additional myocardial protection measures; Incidence of post-aortic clamp-off ventricular fibrillation requiring electrical defibrillation (DC Shock), the total volume of cardioplegia, and the number of doses, cardiopulmonary bypass time, and aortic cross-clamp time, and mortality. In addition, the incidence of atrial fibrillation (AF) and atrial flutter (AFL), mechanical ventilation support time, total ICU stay, and mortality were also assessed postoperatively. All parameters were followed during the ICU stay except mortality was followed up till patient discharge from the hospital. We focused on tracking patient mortality only up to the point of discharge rather than extending through 30 days post-operation, as is standard in the Society of Thoracic Surgeons (STS) guidelines for surgical mortality reporting. This decision was based on several factors specific to our research objectives and constraints. Firstly, our primary aim was to assess the immediate impact of intervention on patient survival, emphasizing the perioperative period and in-hospital outcomes. Moreover, logistical limitations, including the availability of follow-up data and resource constraints, influenced our decision to limit the scope to in-hospital mortality.

### Sample size

To calculate our sample size, we used the differences between the 24-h postoperative troponin levels in del Nido and St. Thomas cardioplegia [20]. The levels were 4.2 (±2.86) and 8.7 (±6.21) ng/mL, respectively. We needed 30 patients in each group to detect a statistical difference in the two-sided test with a 5% alpha error and 90% power. However, we decided to include more patients in this observational study to obtain more reliable results with greater precision and power. Therefore, we chose a sample size of 40 patients for each group.

### Recruitment and allocation

The clinical team identified potential candidates for cardiac surgery either by referral or from the inpatient waiting list. A member of the research team then approached these patients and offered them the to participate in the study. After obtaining consent, eligible patients were randomly assigned to receive either routine del Nido or modified del Nido Cardioplegia Solution once their eligibility was confirmed. This study was approved by the Research Ethical Committee of the School of Medicine-Shiraz University of Medical Science (ID: IR.SUMS.MED.REC.1401.534) and was registered in the Iranian Registry of Clinical Trials (Code: IRCT20230719058845N1).

### Randomization and blinding

The study randomly assigned 120 patients into three groups of 40 each, using block randomization generated via www.sealedenvelope.com in blocks of 6 (6 × 20 = 120) and each block had 20 random codes for either control or Intervention groups. Sealedenvelope.com is a British organization that collaborates with academic institutes and the National Health Service (NHS) of the UK. Their primary focus is to support clinical trials by offering various services through a web-based platform. These services include randomization, allocation concealment, code-break services, and case report management. Sealed Envelope plays a crucial role in supporting clinical trials and ensuring their success [21].

In order to ensure the randomization of the study, codes were generated and sealed in envelopes prior to the day of surgery. On the day of the surgery, perfusionists were entrusted with the responsibility of opening the seals and preparing the cardioplegia solution in accordance with the instructions provided in the randomized codes. This method of code randomization helped to eliminate bias and improve the validity of the study results.

Treatment allocation was concealed from study personnel and participants. The intervention type was blinded by the surgical team, patients, anesthetists, nurses, and laboratory staff. The post-operative care was provided by a specialized team, also blinded to the intervention type. Quality control was performed on data entry for consistency and accuracy.

### Follow-up assessment

The routine del Nido and modified del Nido cardioplegic solutions had different baseline solutions. Despite this difference, both solutions were deemed safe, and the outcomes were regularly audited by the lead investigator every five interventions. The study investigators evaluated the possibility of adverse events to prevent any negative consequences for patients. However, administering a single dose was still effective in preserving the myocardium for an extended period during ischemia when using an aortic cross-clamp and during the follow-up phase no patient was excluded or dropped out.

### Statistical analysis

We used different methods to describe the data depending on whether it was a quantitative or qualitative variable. We used mean and SD for symmetrical distributions, while we used median and IQR for asymmetric distributions. Qualitative variables were described using frequencies and percentages. To compare means between groups, we used a one-way analysis of variance with Tukey’s post hoc analysis. For asymmetric data, we complemented it with the Kruskal-Wallis test and the Dunn test. For comparing proportions among groups, we used Pearson’s *X* ^2^ test, if the minimum expected count was less than 5, we used Fisher’s exact test. For repeated measures, we used the repeated measure ANOVA test, complemented by the Bonferroni test. We set the significance level at 5%, and we analyzed the data using SPSS V.25.

## Results

**Figure 1:**
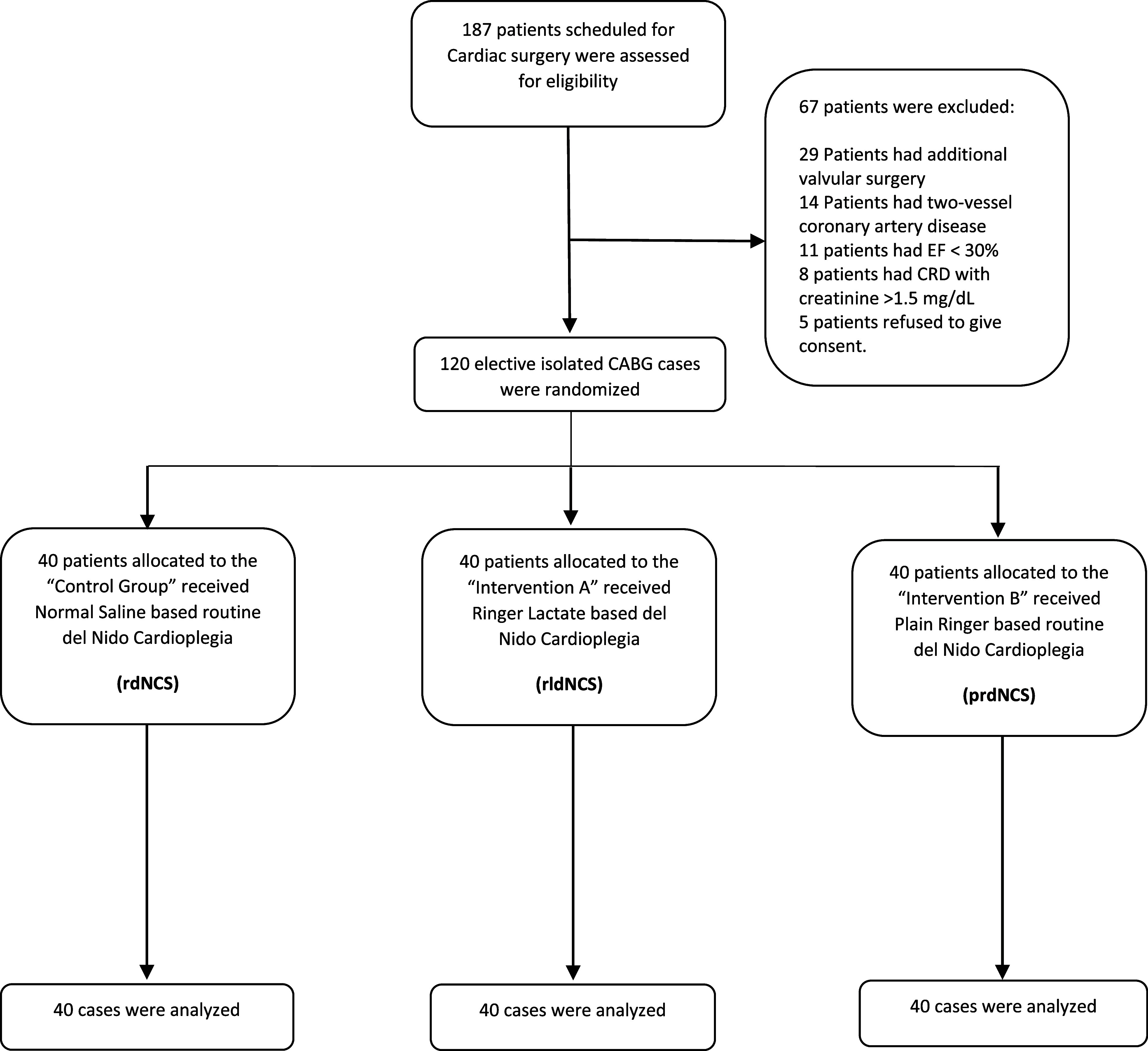
Patient allocation flowchart for control, intervention A and Intervention B groups.

In this study, a total of 120 participants, elected for isolated CABG surgery were included, and randomly allocated into three groups, 40 in each group; Control Group (*n* = 40), Intervention Group A (*n* = 40), and Intervention Group B (*n* = 40) with no potential dropout or exclusion throughout follow-up. The control group received normal saline-based del Nido Cardioplegia Solution (dNCS) also known as rdNCS, Intervention Group A received lactated-based dNCS (rldNCS), while Intervention Group B received plain Ringer-based dNCS (prdNCS) (Figure 1).

The mean age of the rdNCS group was 63 (±6.1), rldNCS group 61 (±10.1), and prdNCS group 62 (±8.7) respectively, and observed with an insignificant difference among the groups; *p* = 0.454. Among rdNCS group 25 (62.5%), rldNCS group 22 (55%), and prdNCS 25 (62.5%) were male and observed with insignificant difference; *p* = 0.732. Mean BMI for the rdNCS group was 26.09 (±4.30), the rldNCS group 25.90 (±4.33), and the prdNCS group 25.35 (±3.67) kg/m^2^ respectively; *p* = 0.732. All clinical characteristics including; Hypertension (HTN), Diabetes Meletus (DM), Hyperlipidemia (HLP), Chronic Obstructive Pulmonary Disease (COPD), Asthma, Pulmonary Hypertension (P.HTN), Carotid Stenosis (C.St), Canadian Cardiovascular Society (CCS) Class 4 Angina, New York Heart Association (NYHA), Ejection Fraction (EF), and EuroSCORE II were normal with no statistical difference among groups respectively; *p* > 0.05 (Table 2).

**Table 2:** Demographic and clinical characteristics.

	Control Group (rdNC)	Group A (rldNCS)	Group B (prdNCS)	*p*-Value
	*n* = 40	*n* = 40	*n* = 40	
Age (y)	63 ± 6.1	61 ± 10.1	62 ± 8.7	0.454
Male	25 (62.5)	22 (55)	25 (62.5)	0.732
BMI (kg/m^2^)	26.09 ± 4.30	25.90 ± 4.33	25.35 ± 3.67	0.732
HTN	25 (62.5)	31 (77.5)	25 (62.5)	0.225
DM	19 (47.5)	17 (42.5)	19 (47.5)	0.874
DM on insulin	6 (15)	7 (17.5)	6 (15)	0.939
HLP	21 (52.5)	15 (37.5)	17 (42.5)	0.388
COPD	0 (0)	2 (5)	0 (0)	0.328
Asthma	3 (7.5)	2 (5)	3 (7.5)	>0.999
P.HTN	2 (5)	2 (5)	1 (2.5)	>0.999
C.St (>50%)	6 (15)	7 (17.5)	14 (35)	0.066
CCS Class 4	3 (7.5)	6 (15)	6 (15)	0.504
Angina				
NYHA				0.309
Class I	14 (35)	11 (27.5)	12 (30)	
Class II	17 (42.5)	12 (30)	9 (25.5)	
Class III	7 (17.5)	12 (30)	13 (22.3)	
Class IV	2 (5)	4 (10)	6 (13)	
EF (%)	50 (45–55)	45 (40–55)	47.5 (40–55)	0.594
Euro SCORE II (%)				0.289
Low risk <4	39 (97.5)	37 (92.5)	34 (85)	
Intermediate risk 4–8	1 (25)	2 (5)	5 (12.5)	
High risk >8	0 (0)	1(2.5)	1 (2.5)	

Frequency with percentage = *N* (%), Mean ± SD and Median (Q1–Q3), BMI (Body Mass Index), HTN (Hypertension), DM (Diabetes Mellitus), HLP (Hyperlipidemia), COPD (Chronic Obstructive Pulmonary Disease), P.HTN (Pulmonary Hypertension), C.St (Carotid Stenosis), CCS (Canadian Cardiovascular Society), NYHA (New York Heart Association), EF (Ejection Fraction).

### Primary outcomes

Primarily the CK-MB, Troponin T, Troponin I, and lactate levels were observed at four different times; post-induction (baseline), 2, 12, and 24 h postoperatively. All four markers were analyzed for three main differences; time effect among the groups, time interactions within the group, and overall differences among the groups based on test values. The study found no significant differences in CK-MB levels based on time effect, time interaction within groups, and among groups based on test values (*p* = 0.344, *p* = 0.787, and *p* = 0.078, respectively). Similarly, no significant differences were observed for Troponin T levels within groups based on time interaction and among groups based on test values (*p* = 0.759 and *p* = 0.143, respectively), but a significant effect for time was found (*p* = 0.034). However, the time difference of Troponin T levels among groups was within the normal range, so it did not support or reject the hypothesis (Table 3).

**Table 3:** Intraoperative and postoperative clinical characteristics.

	Control group (rdNC)	Group A (rldNCS)	Group B (prdNCS)	*p*-value
	*n* = 40	*n* = 40	*n* = 40	
Bypass time (min)	68.5 (60–80)	68.5 (60–86.75)	70 (65–75.75)	0.824
Clamp time (min)	42.5 (35.75–47.75)	41.5 (38.25–54.75)	44.5 (38–49.25)	0.796
Post clamp off VF. DC	14 (35)	11 (27.3)	12 (30)	0.761
Total grafts	4 (3.25–4)	4 (4–4)	4 (3–4)	0.536
CK-MB (U·L^−1^)				
Baseline	12.22 ± 7.26	10.35 ± 7.18	12.82 ± 6.71	Time = 0.344
2 h	13.22 ± 7.09	11.52 ± 7.17	15.30 ± 7.10	Time × Gp = 0.787
12 h	12.05 ± 7.27	11.81 ± 7.29	12.90 ± 7.59	Group = 0.078
24 h	12.61 ± 6.83	11.86 ± 7.32	12.37 ± 7.03	
Trop T (ng·mL^−1^)				
Baseline	0.106 ± 0.055	0.108 ± 0.060	0.105 ± 0.063	Time = 0.034*
2 h	0.128 ± 0.067	0.132 ± 0.060	0.119 ± 0.063	Time × Gp = 0.759
12 h	0.115 ± 0.066	0.133 ± 0.074	0.130 ± 0.066	Group = 0.143
24 h	0.107 ± 0.076	0.126 ± 0.077	0.095 ± 0.069	
Trop I (ng·mL^−1^)				
Baseline	0.016 ± 0.009	0.020 ± 0.016	0.012 ± 0.012	Time = 0.609
2 h	0.015 ± 0.011	0.019 ± 0.015	0.014 ± 0.014	Time × Gp = 0.444
12 h	0.018 ± 0.009	0.020 ± 0.009	0.015 ± 0.014	Group = 0.311
24 h	0.019 ± 0.010	0.017 ± 0.010	0.015 ± 0.015	
Lactate (mg·dL^−1^)				
Baseline	10.42 ± 5.10	11.73 ± 4.89	11.38 ± 4.95	Time = <0.001*
2 h	14.65 ± 6.11	14.03 ± 5.27	12.52 ± 4.60	Time × Gp = 0.064
12 h	15.54 ± 5.61	12.08 ± 5.07	13.30 ± 4.67	Group = 0.129
24 h	12.11 ± 4.30	11.65 ± 4.07	11.15 ± 4.02	
Post operative AFL	2 (5)	3 (7.5)	4 (10)	0.908
Post operative AF	4 (10)	5 (12.5)	4 (10)	>0.999
Ventilation support (h)	5.7 (4.5–6.5)	5.5 (4.1–6.5)	5.7 (4.5–7)	0.580
ICU stay (h)	64 (43.23–85.25)	63.5 (40.50–75.75)	52 (45.50–69.50)	0.484

CK-MB (Creatine Kinase-Myoglobin Binding), Trop T (Troponin T), Trop I (Troponin I), VF. DC (Ventricular Fibrillation needing Direct Current Cardioversion), AFL (Atrial Flutter), AF (Atrial Fibrillation), ICU (Intensive Care Unit).

* *p* < 0.05.

**Figure 2:**
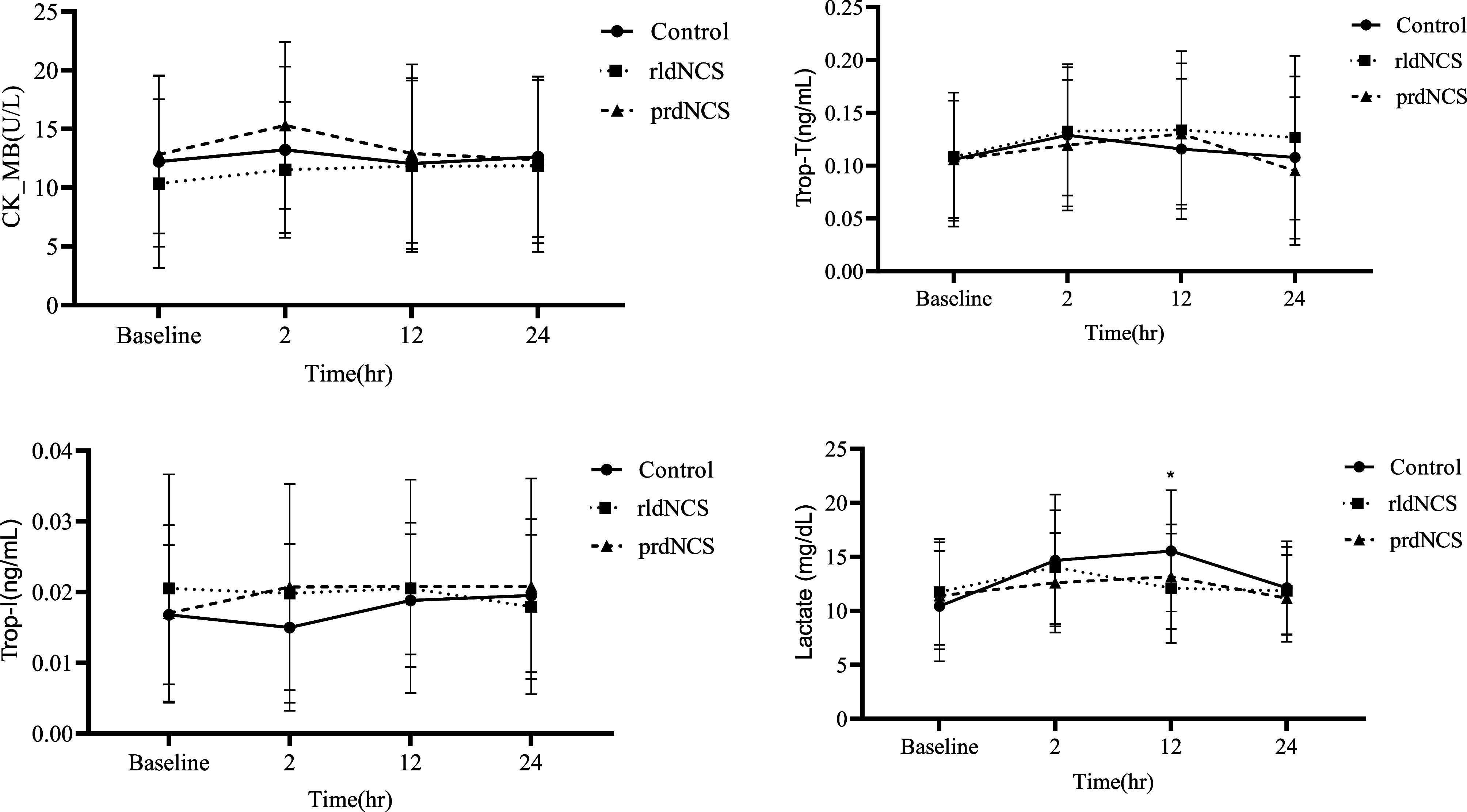
Trends in CK-MB, Troponin T, Troponin I, and lactate levels from baseline to 2 h, 12 h, and 24 h. The control group differs significantly from the rldNCS group (*p* = 0.010).

For Troponin I levels, no significant differences were observed among groups, within groups based on time interaction, and groups interaction based on test values (*p* = 0.609, *p* = 0.444, and *p* = 0.311, respectively). Similarly, no significant differences were observed for lactate levels within groups based on time interaction and groups based on test values (*p* = 0.064 and *p* = 0.129, respectively). However, there was a significant difference among groups based on time effect (*p* < 0.001) and lactate level was high in the control group compared to the Ringer-based del Nido cardioplegia group (*p* = 0.010). Despite this, the time difference in lactate levels among groups was within the normal range, so the difference based on time interaction in lactate levels among groups did not support or reject the hypothesis. See Figure 2 for primary outcomes.

### Secondary outcomes

Secondarily, Intraoperative bypass time, clamp time, cardioplegia volume and number of cardioplegia doses, post-clamp-off ventricular fibrillation needing DC cardioversion, and mortality were observed. Furthermore, postoperative Atrial flutter, Atrial fibrillation, Mechanical Ventilation support, ICU stay and mortality were also observed. The median bypass time for the rdNCS group was 68.5 (60–80), the rldNCS group 68.5 (60–86.75), and the prdNCS group 70 (65–75.75) minutes respectively and there were no significant differences observed among groups; *p* = 0.824. The median clamp time for the rdNCS group was 42.5 (35.75–47.75), the rldNCS group 41.5 (38.25–54.75), and the prdNCS group 44.5 (38–49.25) minutes respectively and there were no significant differences observed among groups; *p* = 0.796. The cardioplegia volume was around 1000 mL and inducted via antegrade in a single dose in each group with no variability. The median number of grafts in the rdNCS group was 4 (3.25–4) the rldNCS group 4 (4–4), and the prdNCS group 4 (3–4) respectively and there were no significant differences observed among groups; *p* = 0.536. However, grafts were not included in the secondary outcomes but to avoid confounding biases we considered the number of grafts as an intraoperative variable too.

In the rdNCS group, 14 (35%) individuals, while in the rldNCS group, 11 (27.3%) individuals, and in the prdNCS group, 12 (30%) individuals were observed with VF needing DC shock cardioversion and there was no significant difference observed among the groups; *p* = 0.761. In the rdNCS group, 2 (5%) individuals, while in the rldNCS group, 3 (7.5%) individuals, and in the prdNCS group, 4 (10%) individuals were observed with AFL postoperatively and there was no significant difference observed among the groups; *p* = 0.908. Similarly, In the rdNCS group, 4 (10%) individuals, while in the rldNCS group, 5 (12.5%) individuals, and in the prdNCS group, 4 (10%) individuals were observed with AF postoperatively and there was no significant difference observed among the groups; *p* > 0.999. The median mechanical ventilation support time for the rdNCS group was 5.7 (4.5–6.5), the rldNCS group 5.5 (4.1–6.5), and the prdNCS group 5.7 (4.5–7) hours respectively and there were no significant differences observed in any of the groups; *p* = 0.580. The median ICU stay for the rdNCS group was 64 (43.23–85.25), the rldNCS group 63.5 (40.50–75.75), and the prdNCS group 52 (45.50–69.50) hours respectively and there were no significant differences observed among the groups; *p* = 0.484 (Table 2). There were no reported deaths till patients were discharged in any of the groups. Additionally, there were no grass harms reported throughout the study.

## Discussion

In recent years, there have been several studies conducted on del Nido cardioplegia and its effectiveness in adult cardiac surgery. However, none of them compared the modified version of del Nido cardioplegia vs other modified versions of del Nido cardioplegia for myocardial protection, which makes this study the first one of its kind as far as we know. One comparative study by Buel, et al. [22] found that del Nido cardioplegia had a six-fold decrease in post-cross-clamp off defibrillation compared to St. Thomas cardioplegia. Another study by Salinas et al. [13] concluded that single-dose del Nido cardioplegia was an effective and economic cardioplegia option with good outcomes in coronary surgery. It was found that most patients had a spontaneous return of sinus rhythm and there was a trend towards decreased transfusion rates. A clinical trial conducted by Niv Ad and his team in 2018 [23] compared whole-blood cardioplegia with del Nido cardioplegia and found that the del Nido could simplify surgical workflow while still being safe and producing similar clinical outcomes and suggested further research to clarify the trend for troponin, which could indicate better heart protection with del Nido solution. In 2019, Kantathut et al. [14] conducted an observational study comparing the efficacy of lactated Ringer’s solution as a base for del Nido cardioplegia vs a standard blood cardioplegia strategy. The results indicated that traditional del Nido cardioplegia ingredients added to lactated Ringer’s provided myocardial protection that was either similar or superior to that of the blood cardioplegia strategy. This suggests that the lactated Ringer solution can be an option for centers that lack access to Plasma-Lyte and should be further investigated.

More recently, several other studies have been conducted to compare different variations of del Nido cardioplegia. Mitsutaka et al. [16] found that normal saline-based modified dNCS can be substituted for the original dNCS solution and still preserve left ventricular function recovery after prolonged global ischemia in piglet models. Karaarslan et al. [15] found that modified del Nido cardioplegia was good in terms of spontaneous sinus rhythm return along with less epicardial edema in their study groups. Tan et al. [17] conducted a meta-analysis study that included 18 Randomized Clinical Trials (RCTs) and 49 observational cohort studies involving 18,191 adult patients and 1634 children. Their findings suggested that del Nido cardioplegia may be associated with lower perioperative mortality than Custodial or Blood Cardioplegia among adult patients undergoing cardiac surgery. However, the risk of atrial fibrillation may be lower with Custodial than with Blood Cardioplegia or del Nido cardioplegia.

Finally, Sevuk et al. [24] compared adult patients undergoing cardiac surgical procedures using tepid normal saline-based modified del Nido cardioplegia vs cold blood cardioplegia patients and found that there was no significant difference between tepid normal saline-based modified del Nido cardioplegia and blood cardioplegia based on intraoperative defibrillation and postoperative peak Troponin T levels. However, mean cross-clamping time, CPB time and total operation time were significantly shorter in the del Nido group. Additionally, Amac et al. [9] conducted a review of articles regarding the use of single-dose Bretschneider cardioplegia and single-dose del Nido cardioplegia in adult cardiac surgery. Their findings showed that del Nido cardioplegia was a superior and safer technique for myocardial protection, resulting in reduced aortic cross-clamp duration, cardiopulmonary bypass time, and required cardioplegia solution volume. This technique also exhibited benefits for many organs and cardiac biochemical parameters.

Our study used a modified version of the del Nido cardioplegia solution as a control group in our setups, which showed no major complications in the past. However, our research found no significant differences in myocardial protection between using normal saline-based, Ringer lactate-based, or plain Ringer-based modified del Nido cardioplegia, alongside other intraoperative and postoperative factors.

Regardless of statistical significance, there were some differences observed, The levels of CK-MB were higher in the plain Ringer-based del Nido cardioplegia group, while Troponin T and Troponin I were higher in the Ringer lactate-based del Nido cardioplegia group, and lactate was higher in the normal saline-based group. Interestingly, the bypass time, clamp time, frequency of atrial flutter, and mechanical ventilation support were higher in the plain Ringer-based del Nido cardioplegia group. After clamping off, VF needing DC shock and ICU stay were higher in the normal saline-based del Nido cardioplegia group, and the frequency of atrial fibrillation was higher in the Ringer lactate-based group. However, since the primary outcome values for all groups were within the normal range, we cannot claim one solution is superior to the others based on these differences. Similarly, based on the various outcomes observed, we cannot confirm our hypothesis that Ringer lactate-based del Nido cardioplegia will prove to be more efficient than the other two groups, in terms of cardiac markers.

Therefore, we concluded that all three solutions, including normal saline, Ringer lactate, and plain Ringer, can serve as a baseline solution for modified del Nido cardioplegia if the Plasma Lyte A solution is unavailable.

## Limitation

Our study had several limitations, but the major one was the use of normal saline as the baseline solution for routine del Nido cardioplegia and considering this modified version as a control in our study. Our results may not be generalizable to other countries with original cardioplegia. Additionally, our inclusion criteria only covered low-risk elective isolated CABG surgeries.

## Recommendation

Lactated Ringer’s solution should be avoided in patients with hypercalcemia, hypokalemia, kidney stones, or a history of metabolic acidosis. Compared to normal saline, it has a lower risk of causing metabolic acidosis due to its lactate content. However, Ringer’s solution may interact negatively with certain medications. Research suggests that intraoperative blood sugar levels increase significantly when Ringer’s lactate is used, especially when combined with dextrose. While Ringer’s lactate is considered superior to normal saline, some clinicians avoid it due to concerns that lactate contributes to hyperglycemia, particularly in diabetic surgical patients. However, lactate in Ringer’s lactate is generally only problematic for patients with liver dysfunction, especially in cases of liver failure.

We recommend a study with a larger sample size and more parameters for primary outcomes to find the best alternative for Plasma Lyte A by keeping the original del Nido cardioplegia as the control standard.

## Conclusion

There were no significant differences in CK-MB, Troponin T, Troponin I, and Lactate levels between using normal saline-based, Ringer lactate-based, or plain Ringer-based modified del Nido cardioplegia, along with other intraoperative and postoperative factors including; bypass time, clamp time, cardioplegia volume, cardioplegia doses, post clamp removal VF, AFL, AF, Ventilation support time, ICU stay and mortality. Therefore, we concluded that all three solutions, including normal saline, Ringer lactate, and plain Ringer, can serve as a baseline solution for modified del Nido cardioplegia in isolated CABG surgery as there was no difference in myocardial protection among them.

## Data Availability

The research data is available on request from the authors.
